# Current View on Major Natural Compounds Endowed with Antibacterial and Antiviral Effects

**DOI:** 10.3390/antibiotics13070603

**Published:** 2024-06-28

**Authors:** Roberto Arrigoni, Andrea Ballini, Emilio Jirillo, Luigi Santacroce

**Affiliations:** 1 CNR Institute of Biomembranes, Bioenergetics and Molecular Biotechnologies (IBIOM), 70124 Bari, Italy; 2 Department of Clinical and Experimental Medicine, University of Foggia, 71122 Foggia, Italy; andrea.ballini@unifg.it; 3 Interdisciplinary Department of Medicine, Section of Microbiology and Virology, School of Medicine, University of Bari “Aldo Moro”, 70124 Bari, Italy; luigi.santacroce@uniba.it

**Keywords:** antibacterials, antibiotics, antivirals, bacteria, marine products, plants, viruses

## Abstract

Nowadays, infectious diseases of bacterial and viral origins represent a serious medical problem worldwide. In fact, the development of antibiotic resistance is responsible for the emergence of bacterial strains that are refractory even to new classes of antibiotics. Furthermore, the recent COVID-19 pandemic suggests that new viruses can emerge and spread all over the world. The increase in infectious diseases depends on multiple factors, including malnutrition, massive migration of population from developing to industrialized areas, and alteration of the human microbiota. Alternative treatments to conventional antibiotics and antiviral drugs have intensively been explored. In this regard, plants and marine organisms represent an immense source of products, such as polyphenols, alkaloids, lanthipeptides, and terpenoids, which possess antibacterial and antiviral activities. Their main mechanisms of action involve modifications of bacterial cell membranes, with the formation of pores, the release of cellular content, and the inhibition of bacterial adherence to host cells, as well as of the efflux pump. Natural antivirals can interfere with viral replication and spreading, protecting the host with the enhanced production of interferon. Of note, these antivirals are not free of side effects, and their administration to humans needs more research in terms of safety. Preclinical research with natural antibacterial and antiviral compounds confirms their effects against bacteria and viruses, but there are still only a few clinical trials. Therefore, their full exploitation and more intensive clinical studies represent the next steps to be pursued in this area of medicine.

## 1. Introduction

Bacterial and viral infections still represent threatening diseases, which cause the deaths of millions of individuals per year [1,2]. With special reference to bacterial infections, the discovery of antibiotics has saved millions of people, but the overdose and misuse of antibiotics has led to the emergence of so-called multi-drug-resistant (MDR) bacteria [3,4,5,6]. Consequently, antimicrobial resistance (AMR) has developed, with the transmission from different sources of bacteria resistant to antibiotics to the general population. Moreover, the frequent use of antibiotics for livestock has greatly contributed to AMR [7]. For instance, resistant animal-borne bacteria can infect humans through direct contact with animals, saliva, and feces or bacteria, which may derive from contaminated water and food and polluted air [8]. Consequently, the dissemination of MDR bacteria in hospitals and intensive care units is extremely frequent [9,10]. Furthermore, the non-rationale use of antibiotics in humans may lead to the alteration of the gut microbiota, which, under steady-state conditions, protects from bacteria and food-borne antigens, enhancing mucosal innate and adaptive immunities or producing bacteria-derived metabolites, such as short-chain fatty acids, secondary bile acids, and tryptophan-derived metabolites that confer protection to the host [11,12,13,14,15,16]. There is evidence that microbiota depletion by antibiotics reduces the production of REG IIIγ, with the defective killing of vancomycin-resistant enterococci (VRE) [17]. In summary, the impact of antibiotics on gut microbiota may lead to a predominance of intestinal pathogenic bacteria and loss of bacterial diversity and/or certain bacterial species, increasing the risk of new infections and/or recurrence [18]. For instance, tuberculosis is again spreading around, as MDR *Mycobacterium tuberculosis* strains have become resistant to antibiotics [19].

Viral infections continue to endanger human life and health, with deadly viruses periodically emerging, as in the case of the human immunodeficiency virus (HIV), Ebola virus, and, mostly recently, SARS-CoV-2 [1,2,20]. Apart from vaccine-mediated preventative measures, more and more antiviral drugs have been discovered, but their uses are limited because of different factors, such as cytotoxicity to host cells and drug resistance, which is related to a high mutation rate, high replication rate, large viral load, and the genetic barrier of viruses [21,22].

Nowadays, alternative treatments to antibiotics and antivirals have been explored, mostly employing natural sources of antimicrobials derived from plants and marine organisms. These sources contain many substances with antibacterial activity, thus potentially restoring the clinical use of antibiotics, increasing their effectiveness while avoiding antibiotic resistance (AR) [23,24]. Parallelly, antiviral compounds of vegetal and marine derivations have been shown to inhibit virus survival and reproduction by targeting enzymes necessary for the replication cycle [25]. In terms of quantities, natural products are often less available, and more research is needed to increase the extraction of bioactive compounds in larger amounts [26].

In the present review, various compounds of natural origin, such as polyphenols, alkaloids, lanthipeptides, and terpenoids, will be described in terms of their antibacterial and antiviral mechanisms of action. Despite a vast arsenal of natural antimicrobials, clinical studies are still scant, while preclinical research still needs to be pursued for a full exploitation of these compounds.

## 2. Natural Products with Antibacterial Activity

Antibiotics still represent the optimal therapeutic approach to combat bacterial infections. However, the phenomenon of bacterial resistance is increasing with the adaptation and survival of bacteria, despite the presence of antibiotics in the environment [26]. Multiple factors contribute to bacterial resistance to antibiotics, and, among them, inappropriate use and dosage (e.g., in viral infections), bacterial carriage, and high concentrations of antibiotics in the environment are the major ones [27]. Notably, the emerging resistance of bacteria to certain antibiotics, such as carbapenems, glycopeptides, and colistin, have been reported [28,29,30,31]. 

Different mechanisms responsible for bacterial resistance have been documented, i.e., the inhibition of some antibiotics’ (beta-lactam antibiotics and tetracyclines) penetration into bacterial cells; prevention of fluoroquinolones and tetracyclines from reaching target cells; modification of the antibiotic target site, as in the case of resistance towards beta-lactam antibiotics, fluoroquinolones, macrolides, and glycopeptides; and the production of special enzymes, which inactivate or modify antibiotics (e.g., resistance to beta-lactam antibiotics, aminoglycosides, and chloramphenicol) [32]. Moreover, there is evidence that point mutations and the recombination of genetic material may provoke bacterial resistance through the acquisition and incorporation of free DNA fragments into the genome, the introduction of resistance genes by a bacteriophage, and the transfer of plasmid-extra-chromosomal genetic material and the transposon fragment of DNA [33]. In view of the increasing occurrence of bacterial resistance, a priority list of antibiotic-resistant bacteria has been proposed [34]. Such a list includes *Pseudomonas (P.) aeruginosa* and *Acinetobacter (A.) baumannii* being carbapenem-resistant; *Enterobacterales*-producing beta-lactamases being resistant to carbapenems; *Mycobacterium (M.) tuberculosis* complex being rifampicin-resistant; and *Neisseria gonorrhoeae* being resistant to all antibiotics.

**Figure 1:**
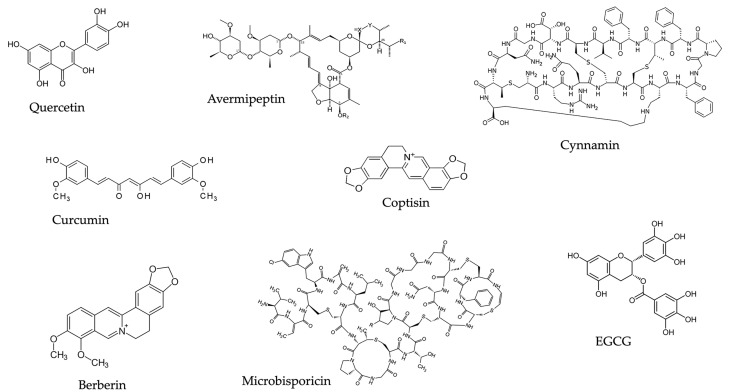
Chemical structures of some natural molecules with antimicrobial activity.

On these grounds, putative alternatives to antibiotics are represented by natural products, which may exert antibacterial activity, restoring the clinical efficacy of classical antibiotics. Plant-derived substances can exert antibacterial activity through multiple mechanisms, such as the alteration of membrane function and structure and blockade of DNA/RNA synthesis and function, as well as interference with cell communication [35]. Furthermore, certain plant-derived compounds can inhibit the production of bacterial toxins. This is the case of essential oils of clove, thyme, cinnamon, and eugenol, which abrogate the production of listeriolysin by *Listeria monocytogenes*, as well as carvacrol, which hampers the production of *Bacillus cereus* and *Clostridioides difficile* toxins [36,37]. Furthermore, certain plant-derived compounds, such as berberine, gallic acid, and capsaicin, can inhibit efflux pumps of bacteria [38]. Figure 1 shows some representative natural products endowed with antimicrobial activity that will be described in the next paragraphs.

Polyphenols like quercetin, curcumin, epigallocatechin gallate (EGCG), lanthypeptides (microbisporicin, cynnamin, and avermipeptin), and alkaloids (berberine and coptisin) are representative of natural products with antimicrobial activity.

### 2.1. Polyphenols

Polyphenols (flavonoids, and non-flavonoids) are largely contained in leaves, seeds, and fruits [39,40]. In general terms, polyphenols are endowed with anti-inflammatory activity, inhibiting the activation of NF-kβ and the release of proinflammatory cytokines, such as IL-1, IL-6, and TNF-α [41]. Moreover, polyphenols in vitro and in vivo expand the T regulatory cell subset with the release of the anti-inflammatory cytokine, IL-10 [41,42]. Quite importantly, polyphenols in vitro hamper the binding of bacterial lipopolysaccharides to toll-like receptor 4 on monocytes, thus interrupting the release of pro-inflammatory cytokines [43]. All together, these activities may alleviate inflammation in infectious diseases of bacterial origin. As far as polyphenol antibacterial activity is concerned, they negatively interact with hydroxyl groups of bacterial cell membranes, thus damaging membrane phospholipids and proteins with expanded membrane permeability and leakage of cell content [41]. Flavonoids, when transformed into pro-oxidants, and phenoxyl radicals can inhibit pathogenic bacteria, causing their lysis [42]. In addition, it has been reported that flavonoids can destabilize cell membranes and cell walls, interfering with bacterial cell attachment [44]. In the next paragraphs, some representative polyphenols are discussed.

Quercetin is a flavonol present in fruits, grain products, and leafy vegetables [45]. It inhibits virulence factors, such as pyogenic proteases and pyocyanin, as well as sialic acid expression, with a decrease in the appearance of quorum-sensing genes [46,47,48]. Furthermore, quercetin has been shown to synergize with antibiotic membrane activity when combined with nanoparticles [46]. There is evidence that quercetin strongly inhibits quorum sensing, biofilm formation, and virulence factors in *P. aeruginosa* and *Staphylococcus aureus* (*S. aureus*) [49]. Synergistic effects have been documented using more than one flavonoid, i.e., quercetin, rutin, and more with some antibiotics, including cefradine, imipenem, ceftriaxone, and methicillin [50]. On the other hand, isoquercetin, a glycosylated flavonoid derived from quercetin, exhibits in subinhibitory concentration (MIC/8) an antagonistic effect in combination with kanamycin, amikacin, neomycin, and gentamycin, in view of mutual chelation [51]. Quercetin, only at a higher MIC, manifests antagonism with antibiotics when applied to the multi-resistant strain of *E. coli.* MICs/MBCs are 50 μg/mL for *S. aureus*, 16–256 μg/mL for *S. mutans*, and 16 μg/mL for MRSA [52,53,54]. Apart from antimicrobial activity, quercetin also exerts a potent anti-inflammatory function, decreasing lipopolysaccharide-mediated effects in *P. gingivalis*-treated human gingival fibroblasts [50]. This effect is attained by the suppression of the NF-kβ pathway and pro-inflammatory cytokine release.

Curcumin, a polyphenolic compound, is a product derived from the stem of curcuma, with a broad spectrum of antibacterial activities against both Gram-negative and Gram-positive bacteria [55,56]. Quite interestingly, curcumin possesses synergistic or additive antibacterial activity in combination with a series of antibiotics, such as polymyxin B, tetracycline, ciprofloxacin, colistin, and other natural adjuvants, i.e., berberine and epigallocatechin gallate (EGCG) [53,54]. Furthermore, curcumin has been demonstrated to inhibit biofilm formation, exerting antimicrobial effects against *P. gingivalis* [55,56]. At the same time, curcumin exhibits anti-inflammatory activity, reducing levels of interleukin (IL)-1β and tumor necrosis factor (TNF)-alpha while increasing the release of the anti-inflammatory cytokine, IL-10 [57].

Catechins (a species of flavan-3-ols) are the main polyphenols of green tea and encompass epicatechin, epigallocatechin (EGC), and EGCG, with EGC and EGCG exerting the most prominent antibacterial activities [58]. Among mechanisms of action, catechins reduce methicillin-resistant *Staphylococcus aureus* (*S. aureus*), inhibiting the N02A efflux pump [59].

Apart from single polyphenols, there exists a series of natural extracts enriched in polyphenols. They include wines and winery by-products (grape pomace, leaves, seeds, and skins), with quercetin, resveratrol, caffeic acid, and gallic acid as main compounds [60]. It has been reported that these extracts are active against a broad spectrum of bacteria, even including *Escherichia coli*, *Salmonella enterica*, *S. aureus*, *Helicobacter (H.) pylori*, *Klebsiella (K.) pneumoniae*, and oral pathogenic bacteria [61]. Antibacterial activity depends on the capacity of these compounds to form pores in the bacterial cell wall, as well as to inactivate microbial adhesion [62]. Also, olive oil by-products contain bioactive compounds, which can contribute to human health [63]. In this respect, olive mill wastewater (OMWW), produced during olive oil extraction, contains hydroxytyrosol as the main polyphenol, with lower amounts of verbascoside and oleuropein [64]. OMWW is active against both Gram-negative and Gram-positive bacteria, which are multi-resistant to antibiotics [65,66]. Of note, OMWW is endowed with anti-inflammatory activity, and its dietary supplementation prevents cell death and oxidative damage in rabbits [67].

Elderberry extracts (*Sambucus nigra* L.) are enriched in phenols, flavonoids, and anthocyanins [68]. Aqueous extracts of the elderflowers are active against *S. aureus* and *S. epidermis*, while ethanolic extracts in vitro exhibit antimicrobial activity against *S. aureus* and *Bacillus cereus* [69,70]. In addition, elderflower extracts are more active against Gram-negative and Gram-positive bacteria, in comparison to fruit extracts, which, instead, are more effective against respiratory infections bacteria [71,72].

Walnuts (*Juglans regia* L.) contain flavonoids and anthocyanins, which account for their antibacterial activities [68]. *E. coli*, *P. aeruginosa*, *H. pylori*, and *S. aureus* represent the major targets of walnuts [73,74]. The antibacterial activity of dried walnuts is enhanced by silver nanoparticles [75]. Honey is a natural supersaturated sugar solution produced by honeybees and is highly enriched in flavonoids and phenolic acids [76]. It exhibits antibacterial activity against both Gram-negative and Gram-positive bacteria, extended-spectrum beta-lactamase-producing *E. coli*, ciprofloxacin-resistant *P. aeruginosa*, and vancomycin-resistant *Enterococcus* (VRE) [77]. Manuka honey, for its contents of methylglyoxal and polyphenols, has been shown to prevent biofilm growth [78]. In this framework, it is worth mentioning propolis, a resin-like material made by bees, rich in polyphenols, phenols, and steroids [79]. There is evidence that flavonoids and cinnamic acid derivatives inhibit bacterial development and adhesion [80].

### 2.2. Essential Oils

Essential oils (EOs) contain volatile and aromatic compounds, as well as phenols in smaller amounts. EOs can modify the structure of bacterial cell membranes, interfering with enzyme and protein functions and with fatty acid metabolism [81]. *Syzygium (S.) aromaticum*, known as clove, belongs to the Myrtaceae family and contains eugenol, a phenyl propanoid, the most bioactive molecule [82]. Eugenol from *S. aromaticum* is active against both Gram-negative, and Gram-positive bacteria while synergizing with the antibiotic colistin against two resistant strains, namely *A. baumannii* and *K. pneumoniae* [83,84]. The combination *S. aromaticum* EOs/eugenol is very active against *P. gingivalis*, killing bacteria after 4 h of treatment and inhibiting the initial step of biofilm formation, while its effect on the pre-existing biofilm is negligible [85].

### 2.3. Alkaloids

Among alkaloids, berberine is a plant metabolite contained in leaves, stems, twigs, barks, rhizomes, and roots of many medicinal plants. It belongs to the group of isoquinoline alkaloids, and is used to synthesize several bioactive molecules [86]. Among different biological activities exerted by berberine, its antimicrobial properties have intensively been studied [87]. Berberine nanoparticles (BRBNPs) have been demonstrated to be very effective in in vitro assays against both Gram-negative, and Gram-positive bacteria. In addition, BRBNPs, when complexed with EGC, were very effective against MRSA in an in vivo murine model [88]. The above complex can affect the ability of MRSA to create a biofilm, inhibiting agrA-D gene expression [89]. In this respect, nanoparticles based on the combination of berberine with cinnamomic acid can more easily penetrate MDR bacteria, thus decreasing biofilm formation [80]. In the same direction, fusic acid, curcumin, and thymol, respectively, when combined with berberine, synergizes in the inhibition of *S. aureus* biofilm formation [90,91,92,93].

Berberine has been shown to be very active against *K. pneumoniae* strains, synergizing with certain antibiotics, i.e., norfloxacin, ciprofloxacin, and doxycycline [94]. Moreover, berberine can restore susceptibility to antibiotics (tigecycline, meropenem, ciprofloxacin, and sulbactam) against multi-drug-resistant *A. baumannii* [95]. Berberine can destabilize the bacterial cell membrane, intercalating and cleaving the bacterial DNA [96]. Moreover, the combination of berberine/thioridazine/ciprofloxacin can reduce the adeABC efflux pump in MDR *A. baumannii* [97]. As far as *E. coli* is concerned, berberine aqueous extract is able to synergize with the antibiotics colistin, tigecycline, and amoxicillin-clavulanate against carbapenem-resistant *E. coli* infections [98]. Also, berberine is an effective antimicrobial against enterotoxigenic and enteropathogenic *E. coli* strains in infected animals [99]. In vitro and in silico studies have demonstrated the efficacy of berberine as a potential efflux pump inhibitor against MdfA from *E. coli* [100].

Regarding *P. aeruginosa*, there is evidence that berberine synergizes with different antibiotics, such as amikacin, azithromycin, and tobramycin, against aminoglycoside-resistant *P. aeruginosa* strains [101,102,103]. It has been reported that berberine can act through blockage of the MexXY-OprM efflux pump, reducing biofilm formation [104,105].

### 2.4. Lanthipeptides

Lanthipeptides are microbial bioactive compounds mostly derived from *Actinobacteria* [106]. The mechanism of action of lanthipeptides is based on their ability to bind to lipid II, a highly conserved peptidoglycan structure in bacterial cytoplasmic membranes [107]. Binding to lipid II leads to pore formation in the Gram-positive bacterial cell membrane, with the release of cellular content [108].

Microbisporicin is a class I lanthipeptide produced by *Microbispora* sp. [109]. Microsporicin is active against a broad spectrum of bacteria, including MRSA, VRE, and penicillin-resistant *S. penumoniae*, as well as *Nisseria meningitidis*, *Moraxella catarrhalis*, and *Haemophilus influenzae* [110,111]. Microbisporicin synergistically acts in combination with the antibiotic polymyxin against Gram-negative bacteria and in murine infection models induced by drug-resistant Gram-positive bacteria. The microbicidal activity of microbisporicin is determined by an increased net charge from halogenation in the lanthipeptide structure, which leads to increased cellular permeability [112]. Microbisporicin is at preclinical stages, however, and further studies are required for its application to infections caused by multi-resistant pathogens [113].

Class II lanthipeptides encompass the cinnamycin group (duramycin, cinnamycin, mathermycin, and kyamycin) [114]. They act by binding to the phosphatidylethanolamine receptor, a major lipid component of the cellular membrane of Gram-positive bacteria [115]. Avermipeptin B belongs to class III lanthipeptides, and it is very active against *S. aureus* [116].

In Table 1, the main natural antibacterial products are depicted.

## 3. Natural Product-Mediated Prevention of Biofilm Formation

Biofilm formation has been shown to play a major role in AR, and therefore, in the next paragraphs, some details on its structure and function will be provided. Biofilm formation encompasses four steps, namely, attachment, microcolony formation, maturation, and dispersion [117]. Extracellular polymeric substance (EPS) matrix formation allows bacterial adhesion, which facilitates the distribution of nutrients to resident cells [118]. EPS acts as a physical barrier, which impedes the penetration of antibiotics, with exopolysaccharides from *P. aeruginosa* binding to cationic antibiotics, such as aminoglycosides [119]. Quite interestingly, in the context of biofilms, there exist so-called persister cells, which are highly tolerant against antibiotics [120]. In a model of S. epidermis biofilm, the importance of persister cells in the development of tolerance to antibiotics has been documented [121].

In the previous sections, various natural products were described for their ability to prevent biofilm formation through different approaches [122]. Here, more emphasis will be placed on marine products for their capacity to target biofilms. Pontifactin, a lipopeptide produced by the marine bacterium *Pontibacter korlensis*, can inhibit the growth of various biofilm formations generated by some bacterial strains, e.g., *B. subtilis, S. aureus*, and *Vibrio (V.) cholerae* [123].

**Figure 2:**
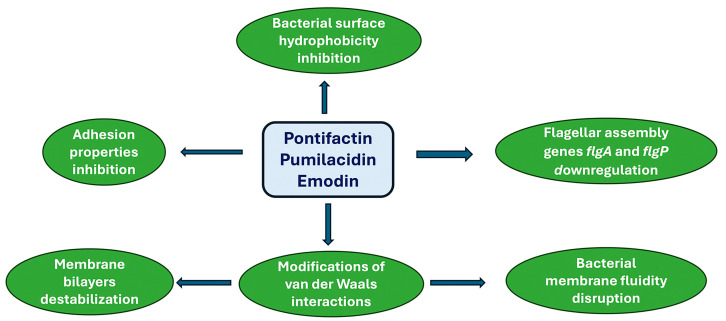
Marine natural product-mediated prevention of biofilm formation.

Lipopetides, because of their amphipathic nature, act as surfactants, reducing the adhesion properties and bacterial surface hydrophobicity [124]. Pumilacidin-like lipopetides, derived from the marine bacterium *Bacillus* sp. 176, inhibits the motility of the biofilm-forming pathogen *V. alginolyticus* 178 [125]. Such an effect is attained through the downregulation of the flagellar assembly genes *flgp* in *V. alginolyticus* 178, which are crucial for motility, flagellar stability, attachment, and colonization. Furthermore, an anthraquinone compound, emodin, isolated from the marine gorgonian coral *Dichotella gemmacea*-associated fungus *Penicillium* sp. SCSGAF 0023, is very effective against *S. aureus* biofilm formation [126]. Evidence has been provided that emodin can penetrate phospholipid bilayers, influencing van der Waals interactions, destabilizing membrane bilayers, and disrupting the fluidity of cell membranes [127]. Other marine peptides have been shown to target quorum sensing. This is the case of the cyclo (L-Trp-L-Pro) isolated from *Rheinheimera aquinaris* QS 102, DKP cyclo (L-Pro-L-Tyr) isolated from *Penicillium chrysogenum* DKY-1, nesfactin isolated from the marine sponge *Fasciospongia cavernosa*-associated bacterium *Nesterenkonia* sp. MSA31, and secalonic acid D, isolated from the marine fungus *Penicillium* sp. SCSGAF0023, respectively, which have been found to inhibit the quorum sensitivity system, preventing the formation of biofilms [128,129,130,131]. In Figure 2, the effects of natural antimicrobials on biofilm formation are summarized.

The antimicrobial activities mediated by major lipopeptides pontifactin (class I, from *Pontibacter korlensis*), pumilacidin (class II, from *Bacillus* sp. 176), and emodin (class III, from *Dichotella gemmacea*) lanthipeptides are described.

## 4. Antiviral Products of Natural Origin

Viruses are small particles (20 to 300 nm in size) containing nucleic acids, proteins, and lipids [132]. They act by attaching to and invading host cells, followed by the removal of the nucleocapsid, the replication of the gene, and final assembly and release [133]. Traditional antiviral drugs inhibit virus survival and reproduction, targeting the enzymes necessary for replication [134]. However, antiviral drugs are cytotoxic while inhibiting viruses, thus causing side effects. Moreover, in view of their proliferation in host cells, high replication rate, large viral load, and genetic barrier, viruses may cause drug resistance [21]. In this regard, the emergence of drug-resistant virus variants has prompted research on more active antiviral compounds. Therefore, new antivirals have been approved, and many of them are substances derived from natural products [135].

### 4.1. Polyphenols

Curcumin is an epigenetic regulator that decreases the expression of histone deacetylase (HDA)C1, HDAC3, and HDAC8 proteins, as well as histone acetyl transferase p300, while enhancing the acetylation of the Ac-histone H4 protein [136]. Curcumin can reduce the amount of hepatitis B surface antigens (HBsAg) and the number of copies with the inhibition of hepatitis B virus (HBV) replication and a decrease in the acetylation level of cccDNA-bound histones H3 and H4 [136]. Moreover, curcumin-mediated downregulation of miRNAs can interfere with certain signal pathways, e.g., Wnt, NF-kβ, MAPK, inflammatory gene responses, and ultimately, viral transmission [137]. Of note, Curcuma longa extracts possess anti-HBV activity, inhibiting the transcription of the HBVX gene A p53-mediated pathway [138]. Evidence has been provided that curcumin can interfere with the binding activity activator protein 1, thus decreasing the transcription of human papilloma virus (HPV)-18 genes [139]. Quite interestingly, curcumin exhibits a potent anti-inflammatory activity in COVID-19 patients, who underwent a higher viral clearance in comparison with the control counterpart [140]. EGCG exerts antiviral activity, inhibiting the replication of many viruses, including the influenza A virus (IAV), HBV, HCV, HSV-1 and HSV-II, HPV, Zika virus, and COVID-19 [141,142]. For instance, EGCG exhibits anti-HCV activity, increasing the expression of miR-548M while decreasing the expression of CD81, the receptor for HCV infection, thus preventing its entry into the hepatocytes [143]. On the other hand, EGCG inhibits miR-122, which is required for HCV replication into the liver [144]. Quite interestingly, EGCG, as well quercetin, upregulates the expression of let-1, thus increasing the expression of interferon, ultimately inhibiting IAV infection [145]. Clinically, a topical ointment with 15% green tea extracts, approved by the US Food and Drug Administration, is currently used to treat genital warts caused by HPV infection [146].

Quercetin induces epigenetic modifications, enhancing histone H3 acetylation via *FasL* overexpression, activation of histone acetyl transferase (HAT), and inhibition of HDAC activities [147]. Additionally, it reduces the expression of miR-146a, which, in turn, acts as a regulator of HIV replication and of NF-kβ signaling [148]. Other antiviral activities exerted by quercetin include the modulation of DNA methylation and histone acetylation, as well as the activation of SIRT-1 [149,150].

Resveratrol, a non-flavonoid, is endowed with antiviral activity as well. In vitro, it inhibits MERS-Cov and SARS-CoV-2, as well as vesicular stomatitis virus replication [151,152]. There is evidence that resveratrol regulates TLR3 expression, inhibiting the TIR domain containing the adaptor molecule pathway and inducing M2 receptor expression, with decreased respiratory syncytial virus (RSV) infections [153]. In addition, resveratrol stops the replication of RSV in human bronchial epithelial cells, activating SIRT-1 and upregulating the release of TNF-α[150]. With the same mechanism, resveratrol can stop HBV infection [154].

### 4.2. Terpenoids

Terpenoids are mainly found in *Thymelaeceae* and *Euphorbiaeceae* and exert anti-HIV activity, activating protein kinase and downregulating HIV-1 cellular receptors [155,156,157]. Research is mostly focused on stelleralide A from the roots of *Stellera chamaejasme* using MT4 cells, and this compound shows potent anti-HIV activity (EC90 of 0.4 nM), with low cytotoxicity (IC50 of 4.3 µM) [157,158,159,160]. The presence of a C-2’ OH group may account for the anti-HIV activity.

The roots and rhizomes of *Valeriana jatamansi* contain valeransin E, which exerts anti-IAV activity, as well as aglycones derived from *Lyonia ovalifolia* and bark extracts of *Burkea africana* [158,159,160]. The antiviral effects of these compounds may be related to the inhibition of viral HA, thus impeding viral entry.

### 4.3. Alkaloids

Berberine is a plant alkaloid that exerts antiviral activity against human cytomegalovirus [161]. It inhibits the progression of the viral cycle at the stage prior to viral DNA replication, interfering with the transactivating functions of the viral immediate early 2 protein, thus hampering early gene expression. Isoquinoline alkaloids, including berberine, isolated from *Coptis chinensis* are shown to interact with the neuraminidase of IAV, exhibiting antiviral activity, compared to oseltamivir and zanamivir [162]. Coptisine, an isoquinoline alkaloid, in silico is the most effective in the inhibition of the main proteases of SARS-CoV-2 [163]. Also, proberberine alkaloids exhibit anti- SARS-CoV-2 activity, preventing the attachment of viral spikes to the ACE2 receptor on host cells [164]. Table 2 illustrates a selection of natural antiviral compounds and their mechanisms of action.

Despite the demonstrated antiviral ability of natural products, they are at the stage of preclinical research, and therefore, their mechanisms of action and side effects (e.g., generation of bacterial resistance) are still unclear [27].

## 5. Discussion

Bacterial and viral infections continue to pose a significant threat to human health worldwide. The ability of bacteria to adapt and develop new resistance mechanisms against antibiotics necessitates novel treatment approaches. Flavonoids, terpenes, alkaloids, and lanthipeptides derived from plant and marine sources have recently been explored as potential antibacterial agents. These natural compounds often demonstrate enhanced effectiveness when used in combination with conventional antibiotics, offering a promising strategy to combat antibiotic resistance. Various mechanisms of action are exerted by natural compounds, and among them, alteration of the bacterial membrane permeability with the formation of micropores and the release of cellular content, as well as the inhibition of the bacterial efflux pump, have been documented. Despite their potential, the clinical application of these natural products faces several challenges. The content of active metabolites in natural extracts is often low, making large-scale extraction difficult. Furthermore, the chemical structure of these compounds can be unstable, necessitating modifications that may alter their activity. More comprehensive studies are required to understand their mechanisms of action fully and to evaluate their safety and efficacy in humans.

Similar to their antibacterial counterparts, natural antiviral compounds have shown promise against a variety of viruses, including HBV, HCV, HIV, IAV, HSV, and SARS-CoV-2. Despite encouraging results obtained in in vitro and in animal models, there are some limitations for their use as antivirals in humans. For instance, the content of active metabolites is very low, and advancements in extraction and purification techniques are essential to obtain higher yields of active metabolites. Furthermore, the structure of natural products is unstable, and therefore, it is necessary to modify it, reconsidering the activity after modification has occurred. Finally, mechanisms of action of natural antivirals have not been clarified in a complete way, and comprehensive preclinical and clinical studies are needed to elucidate the mechanisms of action, optimal dosages, and potential side effects of these natural products.

Moreover, interdisciplinary research is crucial for developing novel formulations and delivery systems that maximize the therapeutic potential of natural compounds. Collaborative efforts can also facilitate the identification of new bioactive compounds from underexplored natural sources, expanding the arsenal of available antimicrobial agents.

Quite interestingly, some molecules (e.g., polyphenols) exhibit multiple biological activities in addition to their antimicrobial effects, such as antidiabetic or anticancer effects. Whether these activities may potentiate or negatively interfere with each other remains an open issue. For instance, polyphenols play an immunosuppressive role in the host, and this may be detrimental to patients with cancer.

## 6. Conclusions

In conclusion, natural compounds offer a vast and largely untapped resource for developing new antibacterial and antiviral therapies. While promising results have been obtained in preclinical studies, significant challenges remain in translating these findings into clinical practice. Addressing these challenges through innovative research and collaborative efforts will be key to unlocking the full potential of natural products in combating infectious diseases.

## Figures and Tables

**Table 1:** Plant-derived compounds and their antimicrobial activity.

Class	Molecule	Biological Action
Polyphenols	Quercetin	Inhibition of quorum-sensing genes, pyogenic proteases, pyocyanin, sialic acid expression, biofilm formation; synergism with antibiotics
	Curcumin	Inhibition of biofilm formation; synergism with antibiotics, berberine, EGCG
	Catechins	Inhibition of the NorA efflux pump
Essential Oils		Modification of cell membrane structure; interference with enzymes, proteins functions, and fatty acid metabolism; synergism with antibiotics
Alkaloids	Berberine	Inhibition of biofilm formation; synergism with antibiotics and other natural products
Lanthypeptides	Class I (Microbisporin)	Pore formation and increased permeability of bacterial cell membrane
	Class II (Cinnamycin)	Binding to the phosphatidyl ethanolamine receptor, a major lipid component of Gram-positive bacteria
	Class III (Avermipeptin B)	Antibacterial activity against *S. aureus*

**Table 2:** Natural antivirals.

Class	Molecule	Antiviral Activity
Polyphenols	Curcumin	X HDAC1, HDAC3, HDAC8, and histone acetyl transferase p300 inhibition; HBsAg and acetylation level of cccDNA-bound histones H3 and H4 inhibition; HBV X gene A p53-mediated pathway transcription inhibition; HPV-18 genes (interfering with the binding activity of activator- protein 1) transcription inhibition; enhancement of the Ac-histone H4 protein; anti-inflammatory activity in patients with COVID-19
Polyphenols	EGCG	CD81 decreased expression, miR-122 inhibition, and suppression of liver HCV replication; anti-HCV activity through increased miR-548m expression
Polyphenols	Quercetin	Inhibition of miR-146a Abd reduced the replication of HIV; modulation of DNA methylation, histone acetylation, and SIRT1 activation
Polyphenols	Resveratrol	TIR domain containing adaptor molecule signaling pathway inhibition, with the induction of M2 receptor expression and decreased RSV replication; regulation of TLR3 expression, SIRT1 activation, and TNF-α release upregulation, with HBV infection inhibition
Terpenoids	Stelleralide A	Inhibition of HIV
Terpenoids	Valeransin E	Inhibition of viral HA
Alkaloids	Berberine	Inhibition of early gene expression; berberine-mediated inhibition of SARS-CoV-2 spike binding histone to ACE2 host cell receptors
Alkaloids	Coptisine	Coptisine-mediated inhibition of SARS-CoV-2 main proteases; berberine-mediated interaction with IAV neuraminidase

## Abbreviations

AMR	Antimicrobial resistance
BRBNPs	Berberine nanoparticles
EGC	Epigallocatechin
EGCG	Epigallocatechin gallate
EOs	Essential oils
EPS	Extracellular polymer substance
HADC	Histone deacetylase
HAT	Histone acetyl transferase
HBV	Hepatitis B virus
HCV	Hepatitis C virus
HIV	Human immune deficiency virus
IAV	Influenza A virus
IL	Interleukin
MDR	Multi-drug-resistant
MSRA	Methicillin-resistant *S. aureus*
OMWW	Olive mill wastewater
RSV	Respiratory syncytial virus
TNF	Tumor necrosis factor
VRE	Vancomycin-resistant Enterococcus
